# Maternal and health service predictors of postpartum hemorrhage across 14 district, general and regional hospitals in Thailand

**DOI:** 10.1186/s12884-020-2846-x

**Published:** 2020-03-18

**Authors:** Phat Prapawichar, Ameporn Ratinthorn, Ketsarin Utriyaprasit, Chukiat Viwatwongkasem

**Affiliations:** 1 Faculty of Nursing Science, Mahidol University, Bangkok, Thailand; 2 Faculty of Nursing, Mahidol University, Bangkok, 10700 Thailand; 3 Department of Biostatistics, Faculty of Public Health, Mahidol University, Bangkok, 10400 Thailand

**Keywords:** Postpartum hemorrhage, Risk factors of postpartum hemorrhage, Intrapartum care, Complication after delivery

## Abstract

**Background:**

Postpartum hemorrhage (PPH) is a preventable complication, however, it remains being the leading cause of maternal mortality and morbidity worldwide including Thailand.

**Methods:**

A case-control study to examine the risk factors associated with PPH across the hospitals under the Ministry of Public Health in Thailand, was conducted. A total of 1833 patient birth records and hospital profiles including human and physical resources from 14 hospitals were obtained. A multiple logistic regression was used identifing the factors that are significantly associated with PPH.

**Results:**

The results show that the rate of PPH varied across the hospitals ranging from 1.4 to 10.6%. Women with past history of PPH were more likely to have increased risk of having PPH by 10.97 times (95% CI 2.27,53.05) compared to those who did not. The odds of PPH was higher in district and general hospitals by 14 (95% CI 3.95,50.04) and 7 (95% CI 2.27,23.27) times respectively, compared to regional hospitals. The hospitals which had inadequate nurse midwife to patient ratio (OR 2.31,95% CI 1.08,4.92), lacked nurse midwives with working experience of 6–10 years (OR 2.35, 95% CI 1.41,3.92), as well as inadequate equipment and supplies for emergency obstetric care (OR 6.47, 95% CI 1.93,21.63), had significantly higher incidence of having PPH, respectively.

**Conclusions:**

This study provides interesting information that the rate of PPH varies across the hospitals in Thailand, in particular where essential nurse midwives, equipment, and supplies are limited. Therefore, improving health care services by allocating sufficient human and physical resources would contribute to significantly reduce this complication.

## Background

Postpartum hemorrhage (PPH) is a leading cause of maternal mortality and morbidity worldwide [1]. The incidence of PPH varies worldwide, with the highest rates found in low-income countries [2], and with rates varying from 1.5% to 22.0% [3]. In Thailand, previous research studies revealed the rates of PPH range from 2.4% [4] to 4.35% [5]. Moreover, in the year 2016, the Ministry of Public Health (MOPH) of Thailand pointed out that some health regions reported maternal deaths caused by PPH up to 50%. Although the MOPH has clearly stated in its national policy that the rate of postpartum hemorrhage should be less than 2.5%, this complication still remains across the hospitals throughout the country. Therefore, identifying the risk factors of PPH is crucial to reducing the rates of this complication.According to the literature, the risk factors for postpartum hemorrhage can be categorized into two main risk factors, including maternal and health service factors [6]. Maternal factors such as age, parity, health status, and obstetric conditions have been associated with increased rates of PPH. For example, research has shown that maternal age of greater than 35 years [7], and multiparous [8] are higher risks for PPH. Moreover, complications during pregnancy, such as pregnant women with diabetes mellitus [9] and pregnant women with chronic anemia [6, 10] appear to also increase the risk of PPH. Women with a past history of PPH were more likely to experience PPH than others [11]. Obstetric factors are another compounding factors that increase the risk for PPH; such as multiple pregnancy [12, 13] and the baby weight of 4000 g or more [14].

Health service factors also influence the rates of PPH including, physical resources (characteristics of the hospital, equipment and supplies) and human resources (health care providers). There is evidence that hospitals with number of deliveries < 200 cases per year had higher odds of PPH than in high-volume delivery hospitals [15] and that some hospitals could not provide emergency obstetric care (EmOC) as they lacked equipment and supplies [16, 17]. Moreover, it has been shown that a larger number of patients to nurse ratio, increased the incidence of patients adverse health outcomes [18, 19]. A research study suggested the rates of mortality increased when the nurse midwives had less than 1 year experience compared to those who had 5 to 9 years of experience, and the rate was significantly decreased when experience increased [20]. Thus, health service factors in term of hospital levels, sufficiency equipment and supplies and the experience of nurse midwives might be associated with the rates of PPH across the hospitals in Thailand.

The objective of this study was to examine the risk factors associated with PPH across the district, general, and regional hospitals under the Ministry of Public Health, Thailand. With previous research studies focused on maternal risk factors associated with PPH, however, there is still limited research available on the associated risk factors of PPH regarding maternal factors and health service factors.

## Methods

### Study design and setting

#### Research design

A case-control study was conducted to examine the maternal and health service risk factors associated with PPH in hospitals across Thailand. The data collection was carried out in 14 hospitals under the Ministry of Public Health (MOPH) throughout the public health area four (The MOPH has divided health service delivery system into 13 public health areas) from October 2015 to September 2016.

#### Setting

**Fig. 1:**
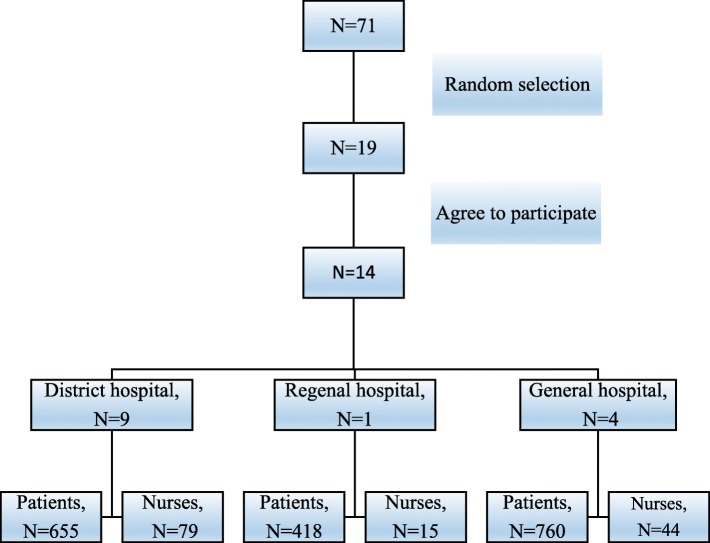
The number and type of hospital settings for data collection

The infrastructure of health care system in Thailand is administered at the national level through the MOPH. The hospital levels are divided into three levels including primary health care level (health care center), secondary health care level (large community or district and general hospitals) and tertiary health care level (tertiary and regional hospitals). The hospital levels that have the capacity and facilities of health care providers, equipment, and supplies for women during intrapartum period include secondary and tertiary health care levels. The MOPH has divided health service delivery system into 13 public health areas including Bangkok. Each public health area consists of four to eight provinces (except Bangkok). The public health area four which includes eight provinces that were selected as purposive sampling in this study. There are 71 hospitals under the MOPH in these provinces which have the facilities to provide care for women during intrapartum period. The steps for sample selection of the hospital are described as follows:

The distribution of PPH was classified into two groups according to the severity of the rate of postpartum hemorrhage, as well as lower and higher than national labor room quality indicator ≤2.5% and >  2.5%, respectively. There were 38 out of 71 hospitals reported the rates of PPH 0.1% and more (21 hospitals had rates of PPH 0.1 to 2.5%) and 17 hospitals had rates of PPH higher than the national quality indicator level. In order to achieve a good representative of the samples, thus, 19 out of 38 hospitals were randomly selected for data collection by using simple random sampling. However, 14 hospitals agreed to participated in the study as presented in Fig. 1. All participated hospitals used transparency plastic bag for estimation of blood loss after vaginal delivery. The incidence of PPH was diagnosed when women had blood loss of 500 ml or more within 24 h after vaginal delivery.

### Study participants

All women who delivered during the time frame of the study period were included. The inclusion criteria for this study were women who gave birth vaginally. Exclusion criteria included; 1) women who underwent cesarean section (C/S) as this study focused on PPH following vaginal birth during intrapartum period and 2) women who gave birth before admission to the hospital (BBA).

The sample size was calculated using the formula described as shown below [21].

$$ n=\left(\frac{r+1}{r}\right)\frac{\left(\overline{p}\right)\left(1-\overline{p}\right){\left({Z}_{\beta }+{Z}_{\alpha /2}\right)}^2}{{\left({p}_{1-}{p}_2\right)}^2} $$

According to the formula, the total number of cases (*n*) equal 153. In general, if the number of cases is small, the ratio of controls to cases can be raised to improve the significant statistics [22] and a matching ratio of case to control up to 1:4 could produce the lowest bias [23]. The national data base found that the rates of PPH across 71 hospitals throughout the public health, area four, in the year 2016 varied, ranging from 0 to 53 cases or total rates equals to 1.28%. Therefore, to get sufficient number of participants for the data analysis in the “case” group, all cases of PPH following vaginal delivery during the study period were included in the study. However, the statistics regarding the number of delivery per year across the hospitals were different. Thus, the representation of the participants in the control group were randomly selected by using a quota sampling technique. In general, if the number of cases is small, the ratio of controls to cases can be raised to improve the significant statistics [22] and a matching ratio of case to control up to 1:4 could produce the lowest bias [23]. Therefore, to increase the sample size to be adequate to perform analyses with high statistical power, the case to control ratio of 1:10 is used in this study. In addition, the estimated sample for case-control study was approximated to 1683 cases which included women with PPH ie. 153 cases and 1530 cases for non-PPH (controls). Quota sampling technique used in this study; if the total number of women delivered vaginally was less than 1000 and 1000–10,000 cases annually, 25 and 10% were selected as representative number for data analysis, respectively [24].

### Data collection procedures

#### Data collection for maternal factors

The data record form was developed to elicit information about the maternal factors including 1) demographic data: age; 2) reproductive history: parity; 3) pregnancy information: gestational age, complication during pregnancy such as anemia, twin, gestational diabetes mellitus, and past history of postpartum haemorrhage, 4) delivery data: method of delivery, birth assistant, episiotomy, perineum lacerations, using prophylactic uterotonics, duration of each stage of labor, estimate blood lost, baby birth weight, and complication during labor such as retained placenta, poor uterine contractions.

#### Procedure

After getting IRB approval from the participated hospitals, researcher firstly contacted head nurse of labor room for data collection. All birth record for the year of 2015–2016 were accessed and did a randomly select of each case used research article of Viwatwongkasem [24] to get proportion, then, extract data from the file to put in case record form.

#### Data collection for health service factors

There were two parts of the health service factors including 1) general characteristics of the hospital such as number of beds, number of delivery via vaginal or cesarean section per year, and signal functions for emergency obstetric care (EmOC) and 2) general information of health care providers such as number of nurse midwife and patient ratio, working experience, and training for PPH management in the past 1 year. Head nurse per hospital completed the form and verified data by using hospital records and nurse interviews to increase the accuracy of repeated data.

#### Data analysis

This research study aimed to examine the factors associated with postpartum hemorrhage which included maternal factors and health service factors. Standard descriptive statistics was used to summarize women and hospital characteristics (mean, SD, frequencies). All independent variables from the study were analyzed using a univariate model in order to select the independent variables (presented a *P*-value < 0.05) in the best model of multiple logistic regression analysis with enter method. The model testing for predictive factors associated with PPH was performed as follows: the assumption for statistics use of multilevel logistic regression analysis was tested by using intraclass correlation coefficient (ICC) to see the variance of health service level that contributed to postpartum hemorrhage, with an ICC value being greater than 0.1 [25]. Based on the results of the ICC tests for this study, the ICC value was less than 0.1. This indicates that there were no variations of hospital levels but variations of hospital levels were already explained by the independent variables. Therefore, multiple logistic regression was used to estimate odds ratio (OR) and 95% confidence interval (95%CI) for predictive factors associated with postpartum hemorrhage.

## Results

### Women’s characteristics

A total of *N* = 1833 women were included in the analyses. The mean age of participants was 26 years old (SD, ranged 11 to 49). The majority (78.5%) of them were of the gestational age 38–42 weeks. Approximately 35.3 and 64.7% of them were primiparous and multiparous women, respectively. There were 1478 (80.6%) of participants who received episiotomy, whereas 28 (1.5%) out of all participants experienced 3rd or 4th degree perineum lacerations. Average blood lost for vaginal delivery was 287.36 ml, and the overall mean of baby birth weight was 3074.5 g (SD ± 414.28). The distributions of women’s characteristics are illustrated in Table 1.

**Table 1:** The rate and range of PPH

	Patients (%) (*n* = 1613)	None PPH (*n* = 1613)		PPH (*n* = 220)			*P* -Value
		n	%		n	%	
**Age**							0.631
*< 20 years*	376 (20.5)	336	89.4		40	10.6	
*20–34 years*	1241 (67.7)	1089	87.8		152	12.2	
*≥ 35 years*	216 (11.8)	188	87.0		28	13.0	
(Minimum = 11 yr., Maximum = 49 yr., mean = 26 yr., SD ± 6.68)							
**Gestational age**							0.69
*< 38 wks*	395 (21.5)	358	83.2		37	16.8	
*38–42 wks*	1438 (78.5)	1255	77.8		183	22.2	
**Parity**							.897
*Primiparous*	651 (35.5)	527	87.9		79	12.1	
*Multiparous*	1182 (64.5)	1041	88.1		141	11.9	
**Complication during pregnancy**							
Anemia							**< 0.001**
*No*	1688 (92.1)	1499	92.9		114	7.1	
*Yes*	145 (7.9)	189	85.9		31	14.1	
Twin							.255
*No*	1830 (99.8)	1611	88		219	12	
*Yes*	3 (0.2)	2	66.7		1	33.3	
GDM							.096
*No*	1803 (98.4)	1590	88.2		213	11.8	
*Yes*	30 (1.6)	23	76.7		7	23.3	
Past history of PPH							**.003**
*No*	1824 (99.5)	1608	88.2		174	11.8	
*Yes*	9 (0.5)	5	55.6		46	44.4	
**Complication during labor**							
*-Retained placenta*							**< 0.001**
*No*	1781 (97.2)	1607	90.2		174	9.8	
*Yes*	52 (2.8)	6	11.5		46	88.5	
*-Poor ut.contractions*							**< 0.001**
*No*	1725 (94.1)	1606	93.1		119	6.9	
*Yes*	108 (5.9)	7	6.5		101	93.5	
**Method of delivery**							.337
*Normal birth*	1763 (96.2)	1555	88.2		208	11.8	
*F/E*	9 (0.5)	8	88.9		1	11.1	
*V/E*	61 (3.3)	50	82		11	18	
**Birth assistant**							0.45
*Obstetricians*	202 (11.1)	174	86.1		28	13.9	
*Nurse midwives*	1617 (88.7)	1426	88.2		191	11.8	
*Medical students*	5 (0.3)	4	80		1	20	
*Nursing students*	4 (0.2)	4	100		0	0	
**Episiotomy**							.308
*Yes*	1478 (80.6)	1295	87.6		183	12.4	
*No*	355 (19.4)	318	89.6		37	10.4	
**Perineum lacerations**							**< 0.001**
*Intact Perineum*	377 (20.6)	356	94.4		21	5.6	
*First Degree*	332 (18.1)	282	84.9		50	15.1	
*Second Degree*	1096 (59.8)	958	87.4		138	12.6	
*Third and Fourth*	28 (1.5)	17	60.7		11	39.3	
**Using uterotonic drugs**							.39
*Yes*	1756 (95.8)	15	6.8		205	93.2	
*No*	77 (4.2)	62	3.8		1551	96.2	
**Duration of labor**							
1st stage of labor							.305
*< 10 h.*	1344 (73.3)	1189	88.5		155	11.5	
*> 10 h.*	489 (26.7)	424	86.7		65	13.3	
2nd stage of labor							**< 0.001**
*< 1 h.*	1774 (96.8)	1573	88.7		201	11.3	
*> 1 h.*	59 (3.2)	40	67.8		19	32.2	
3rd stage of labor							**< 0.001**
*< 20 min*	1760 (96.0)	1575	89.5		185	10.5	
*> 20 min*	73 (4.0)	38	52.1		35	47.9	
**Baby birth weight**							**< 0.001**
*< 2500 g*	109 (5.9)	100	91.7		9	8.3	
*2500–3500 g*	1487 (81.1)	1327	89.2		160	10.8	
*> 3500 g*	237 (12.9)	186	78.5		51	21.5	

Birth weight (mean = 3074 g; SD ± 414.28).

*Remarks: GDM* Gestational diabetes mellitus; *PPH* Postpartum hemorrhage; *F/E* Forceps extraction; *V/E* Vacuum extraction.

### Characteristics of health service

#### Hospital characteristics

The findings revealed that there were 22,491 women who used the services in labor room units throughout the year. The number of women who gave birth via vagina and cesarean section (C/S) were 9075 and 5687 respectively. The rate of PPH varied across the hospitals ranged from 1.4 to 10.6%. There were six hospitals that demonstrated the rate of postpartum hemorrhage was higher than national quality indicator of labor room (> 2.5%). The number of health care providers including obstetricians, anesthesiologists, and nurse midwives were 24, 17, and 138, respectively. The results showed that nearly two-thirds (70.8%) of obstetricians provided care in regional and general hospitals, while only 29.1% worked in district hospitals. Nurse midwives were the main group of health care providers that provided obstetric care such as monitoring and conducting delivery for women during intrapartum period.

The results shown in Table 2 indicate that there were variations in equipment and supplies for providing emergency obstetrics care (EmOC) across hospital levels. The general and regional hospitals were more likely to report that they had sufficient equipment and supplies for providing EmOC compared to district hospitals (*p* = 0.001). The findings revealed majority (77.8%) of district hospitals had never performed all eight EmOC signal functions. More than half (66.7%) of district hospitals reported no operation room for caesarean section. Forty-four percent revealed lack of equipment and supplies for performing removal of retained products, with one hospital reporting being unable to perform manual removal of placenta due to lack of equipment and an obstetrician for performing the procedure. For the caesarean section, only 11.1% of district hospitals met the criteria for comprehensive emergency obstetric care (performing blood transfusion and caesarean section). The general and regional hospitals reported that there were no problems regarding equipment and supplies for basic and comprehensive emergency obstetric care as shown in Table 2.

**Table 2:** Availability of equipment and supplies for emergency obstetrics care (EmOC)

Characteristic		Type of hospital				Total (*n* = 14)	
		Region (*n* = 1)	General (*n* = 4)	District (*n* = 9)			
Services provided (EmOC signal functions)							
1. Parental oxytocics		1	4		9	14	
2. Parental antibiotics		1	4		9	14	
3. Parental anticonvulsants		1	4		9	14	
4. Assisted vaginal delivery		1	4		9	14	
5. Removal of retained products		1	4		5	10	
6. Manual removal of placenta		1	4		8	13	
7. Blood transfusion provided		1	4		9	14	
8.Caesarean section		1	4		3	8	

### Factors associated with PPH

In univariate analyses, eight maternal factors including anemia, past history of PPH, baby birth weight > 3500 g, severe perineum laceration, retained placenta, poor uterine contractions, second stage of labor > 1 hour, and third stage of labor > 20 min were significantly associated with PPH (*p* < 0.05), as shown in Table 3.

**Table 3:** Univariate logistic regression analysis of maternal factors associated with PPH (*N* = 1833)

	n (%)	None PPH (*n* = 1613)		PPH (*n* = 220)		*P*-Value	Crude OR (95% CI)
		n	%	n	%		
Anemia							
No	1688 (92.1)	1499	92.9	114	7.1	Ref.	
Yes	145 (7.9)	189	85.9	31	14.1	**0.001**	2.16 (1.37, 3.40)
Past history of PPH							
No	1824 (99.5)	1608	88.2	216	11.8	Ref.	
Yes	9 (0.5%)	5	55.6	4	44.4	**0.01**	5.65 (1.42, 22.47)
Perineum lacerations							
*Intact*	377 (20.6)	356	94.4	21	5.6	Ref.	
*First Degree*	332 (18.1)	282	84.9	50	15.1		1.98 (0.97, 4.05)
*Second Degree*	1096 (59.8)	958	87.4	138	12.6		1.86 (1.01, 3.45)
*Third and Fourth degree*	28 (1.5)	17	60.7	11	39.3	**< 0.001**	9.75 (3.25, 29.24)
-Retained placenta**							
*No*	1781 (97.2)	1607	90.2	174	9.8	Ref.	
*Yes*	52 (2.8)	6	11.5	46	88.5	**< 0.001**	52.25 (19.03,143.46)
-Poor ut.contractions**							
*No*	1725 (94.1)	1606	93.1	119	6.9	Ref.	
*Yes*	108 (5.9)	7	6.5	101	93.5	**< 0.001**	172.69 (76.89,387.85)
Baby birth weight							
< 2500 g	109 (5.9)	100	91.7	9	8.3		0.87 (0.42, 1.78)
2500-3500 g	1487 (81.1)	1327	89.2	160	10.8	Ref.	
> 3500 g	237 (12.9)	186	78.5	51	21.5	**< 0.001**	3.05 (1.44, 6.44)
Duration of labor							
*1st stage of labor*							
< 10 h.	1344 (73.3)	1189	88.5	155	11.5	Ref.	
> 10 h.	489 (26.7)	424	86.7	65	13.3	0.305	1.09 (0.79, 1.51)
*2nd stage of labor*							
< 1 h.	1774 (96.8)	1573	88.7	201	11.3	Ref.	
> 1 h.	59 (3.2)	40	67.8	19	32.2	< 0.001*	3.56 (1.98, 6.41)
*3rd stage of labor*							
< 20 min	1760 (96.0)	1575	89.5	185	10.5	Ref.	
> 20 min	73 (4.0)	38	52.1	35	47.9	< 0.001*	7.69 (4.72, 12.54)

*OR* odds ratio; *CI* confidence interval; * = *P*-values < 0.05, statistical significant at level 0.05, ** = adjusted variables.

There were seven health service factors significantly associated with PPH (p < 0.05), namely hospital levels, inadequate nurse midwife to patient ratio, nurse midwives working experience of less than 6–10 years, workload more than 25 days, ratio of nurse midwives in afternoon and night shifts less than 2 persons, and inadequate equipment and supplies for EmOC. (Table 4).

**Table 4:** Univariate logistic regression analysis of organizational factors associated with postpartum hemorrhage (*N* = 1833)

	n (%)	None PPH (*n* = 1613)		PPH (*n* = 220)		P-value	Crude OR (95% CI)
		n	%	n	%		
**Hospital levels**							
Region	276 (15.1)	259	93.8	17	6.2	Ref.	
General	861 (47.0)	733	85.1	128	14.9		2.00 (1.57,4.49)
District	696 (38.0)	621	89.2	75	10.8	< 0.001*	1.84 (1.07,3.18)
**Having obstetrician**							
No	303 (16.5)	259	85.5	44	14.5	0.140	1.31 (0.92,1.87)
Yes	1530 (83.5)	1354	88.5	176	11.5	Ref.	
**Having the standard of nurse midwife to patient. Ratio**							
No	1613 (88)	157	82.6	33	17.4	**0.016**	1.83 (1.22,2.74)
Yes	220 (12)	1456	88.6	187	11.4	Ref.	
**Having nurse midwife working experience of 6–10 years**							
No	1613 (88)	733	91.3	70	8.7	**< 0.001**	1.83 (1.31,3.38)
Yes	220 (12)	880	85.4	150	14.6	Ref.	
**Workload**							
< 25 days	220 (12)	247	87	37	13	Ref.	
> 25 days	1613 (88)	1366	88.2	183	11.8	**0.009**	2.01 (1.19,3.38)
**No. of Nurse midwives in day shift**							
< 2 persons	1613 (88)	416	87.4	60	12.6	0.638	1.08 (0.79,1.48)
> 2 persons	220 (12)	1197	88.2	160	11.8	Ref.	
**No. of Nurse midwives in evening shift**							
< 2 persons	1613 (88)	822	85.2	143	14.8	< 0.001*	1.79 (1.33,2.40)
> 2 persons	220 (12)	791	91.1	77	8.9	Ref.	
**No. of Nurse midwives in night shift**							
< 2 persons	1613 (88)	822	85.2	143	14.8	< 0.001*	1.79 (1.33,2.40)
> 2 persons	220 (12)	791	91.1	77	8.9	Ref.	
**Adequate equipment and supply for EmOC**							
*No*	1344 (73.3%)	1192	88.7	152	11.3	**0.004**	3.70 (1.54,8.93)
*Yes*	489 (26.68%)	421	86.1	68	13.9	Ref.	

*OR* odds ratio; *CI* confidence interval; * = *P*-values < 0.05, statistical significant at level 0.05.

### The predictive factors associated with PPH

All significant maternal and health service factors variables were analyzed by using multiple logistic regression model. After adjustment, the findings revealed that women who had past history of PPH (OR 10.97, 95% CI 2.27–53.05, *p* = 0.003) and baby birth weight more than 3500 g (OR 1.98, 95% CI 1.19–3.30, *p* = 0.008) were more likely to have increased risk of having PPH. For the health service factors, the results demonstrated that the levels of district hospital (OR 14.06, 95% CI 3.95–50.04, *p* < 0.001) and general hospital (OR 7.27, 95% CI 2.27–23.27, *p* < 0.001) were more likely to increase the odds of having PPH 14 and 7 times respectively compared to regional hospital. The hospitals with inadequate nurse midwife to patient ratio had significantly increased the odds of having PPH two times compared to the hospitals that had adequate nurse midwife to patient ratio (OR 2.31, 95% CI 1.08–4.92, *p* = 0.03). The study found that lack of nurse midwives working experience of 6–10 years in the unit were more likely to increase the risk of having PPH more than two times (OR 2.35, 95% CI 1.41–3.92, *p* = 0.001). The hospitals with inadequate emergency obstetric care resources had significantly increased rate of PPH by 6.47 times compared to the hospitals that had adequate equipment and supply for EmOC (OR 6.47, 95% CI 1.93–21.63, *p* = 0.002) as presented in Table 5. Anemia and baby birth weights were not significantly associated with increased the risk of PPH (OR 2.29, 95% CI .64–2.58, *p* = 0.48) and (OR 2.51, 95% CI .89–7.08, *p* = 0.08) respectively.

**Table 5:** Multiple Logistic Regression Analysis of predicting factors associated with PPH (*N* = 1833)

Factors	OR	95% CI		***P***-value
*Anemia (REF = No)				
Yes	1.29	0.64	−2.58	0.481
Past history of PPH (REF = No)				
Yes	10.97	2.27	−53.05	0.003
*Perineum lacerations (REF = Intact perineum)				
First degree	6.07	2.60	−14.15	0.000
Second degree	5.65	2.63	−12.11	0.000
Third and fourth degree	29.59	8.65	− 101.20	0.000
*Retained placenta (REF = No)				
Yes	71.47	27.37	− 186.65	0.000
*Poor uterine contractions (REF=No)				
Yes	260.11	111.15	−608.70	0.000
Baby birth weight (REF = 2500–3500 g)				
< 2500 g)	0.79	0.30	−2.73	0.635^ns^
> 3500 g	1.98	1.19	−3.30	0.008
Hospital levels (REF = Regional hospital)				
District hospital	14.06	3.95	−50.04	0.000
General hospital	7.27	2.27	−23.27	0.001
Adequate nurse midwife to patient ratio (REF = Yes)				
No	2.30	1.08	−4.92	0.031
Having nurse midwife working experience of 6 to 10 years (REF=Yes)				
No	2.35	1.41	−3.92	0.001
Adequate equipment and supply for EmOC (REF = Yes)				
No	6.47	1.93	−21.63	0.002
Constant	0.00	0.00	−0.00	0.000
-log likelihood	− 369.65			
AIC	769.31			
BIC	852.01			

Remarks: * = Adjusted by the confounder variables including anemia, perineum lacerations, retained placenta, and poor uterine contractions; *EmOC* Emergency Care; *ns* No statistical significance; *REF* Reference; *OR* Odds ratio; *CI* confidence interval; statistical significant at level 0.05.

## Discussion

This study aimed to examine the risk factors associated with PPH by examining both maternal and health service factors across hospital levels under the MOPH throughout eight provinces in Thailand. The results suggest that the rates of PPH varies across the hospitals ranged from 1.4 to 10.6%. This finding is congruent with the previous research studies conducted in Thailand that the rates of PPH varied across the hospitals [4, 5] There are two mains factors associated with PPH were maternal and health service factors. Some characteristics of women are already known as risk factors of PPH, such as age > 35 years old [6], severe perineum lacerations [26], anemia and multiple pregnancy [11]. However, these variables were not predictive factors for PPH in this study except they showed history of PPH and delivered a baby with a weight of more than 3500 g. The findings reveal that those women who experienced PPH are more likely to have increased the odds of PPH than those without, this congruent with previous studies that past history of PPH is associated with PPH [27, 28]. This study reveals that baby birth weight higher than 3500 g significantly increases the rate of PPH. This finding is similar to previous studies that found increase baby birth weight was a significant risk factor of PPH [8, 27].

For the health service system, the result show rates of PPH varied across hospital levels and were commonly found in district hospitals. The reasons for this might be due to lack of equipment and supplies to perform EmOC, such as lack of operating rooms, manual removal of placenta and removal of retained products for example. These results support the need to improve health services system for PPH for some hospitals. This finding was similar to previous published studies where hospital levels appeared to influence resources allocation and performance outcome of health care providers providing effective care [29] as well as PPH management [6]. Although it is clear that sufficient resource allocation can prevent maternal complications after delivery [30, 31], many health care institutions could not provide the standard of EmOC which is recommended by WHO [16, 29, 32, 33].

Moreover, the finding of this study demonstrates that the characteristics of health care providers namely the inadequate nurse midwife to patient ratio and working experience are associated with the high rates of PPH. This study has shown that approximately 71.43% (*n* = 10) of the participated hospitals reported the nurse midwife to patient ratio did not meet the standard criteria (2:1) and less than the recommendation by the Thailand Nursing and Midwifery Council, which significantly increases the risk of PPH. Some hospitals, have only one nurse midwife working at night or afternoon shifts. Therefore, it is difficult for them to provide adequate nursing care or interventions for women during delivery, such as inability to inject uterotonics medicine within 1 min after the baby birth to promote uterine contractions and prevent PPH as recommended by the MOPH Thailand. This might result to poor uterine contractions which may lead to developing PPH after vaginal delivery. Previous studies pointed out the larger number of patients per nurse ratios was significantly associated with higher incidence of adverse events such as wrong medication or dose (odds ratio = 1.01, 95%CI 1.007–1.016), and falls with injury [18]. Increase workload of nurse midwives could also increase the risk of negative health outcomes of women because of difficulties to perform optimal care during intrapartum period [34].

Surprisingly, this study revealed a lack of nurse midwives with working experience of 6–10 years significantly increased the rates of PPH (OR 2.20, 95% CI 1.50–2.23). This finding is consistent with previous study from New Zealand that revealed the rates of perinatal mortality appeared to increase when the nurse midwives had experience less than 1 year compared to those who had 5 to 9 years of experience (rate ratio 1.33; 95% CI 1.02–1.73) and the rates of perinatal mortality was significantly decreased when nurse midwives’ experience is increased (*p* = 0.031) [20]. This might be because those nurse midwives who had working experience in providing care during intrapartum period had developed their skills and shown increased confidence to handle the uncertain situations and were able to manage well the situation during delivery [35] which helped to prevent and reduce the rates of PPH [36, 37]. In this study, 50 % of all participated hospitals lacked nurse midwives who had working experience of 6–10 years, in particular, those in district hospitals (66.7%). These might be possible reasons to explain the rate of postpartum hemorrhage still remained across hospital levels in the public health area four, in particular at the district level.

### Implication for practice

PPH is preventable complication, and the analysis of this study suggest that maternal factors appear to increase the rate of PPH, in particular those who had past history of PPH and delivered baby whose weight more than 3500 g. Therefore, major changes in provision need to be accompanied by utilizing initial screening, appropriate monitoring and evaluation during delivery, that could potentially reduce the rate of PPH following vaginal delivery. The study suggests that lack of nurse midwives working experience in the unit could increase the risk of PPH. Thus, maintaining and assigning experienced nurse midwives for taking care of women during intrapartum period in each shift and promote additional training for obstetrics risk management for less experienced nurse midwives are important factors to improve good health outcomes of women after delivery. Moreover, inadequate nurse midwife to patient ratio and inadequate equipment and supplies for performing EmOC in labor room are classified as predictive factors in this study. These might be the causes of having the highest rates of PPH in district hospital levels. Therefore, sufficient resource allocations for EmOC for women during intrapartum period is recommended. For health service system, supporting the routine use for PPH risk assessment is needed [38]. Moreover, careful monitoring during intrapartum period such as performing initial screening for maternal factors that assess past history of PPH and underlying diseases for example, in order to early detect and prevent the incidence rates of PPH [39] and also to ensure the quality of care is met.

### Limitations

The findings of this research study show the rate of PPH across 14 hospitals under the MOPH in Thailand throughout the public health area four only and could not be used as the representative sample of the country. The rate of PPH in this study included only women who underwent vaginal delivery. The distribution and risk factors of PPH across the hospitals types throughout the country should be investigated.

## Conclusion

The findings pointed out that the distribution of PPH are varied across the hospital levels. There were two factors significantly associated with PPH including maternal factors and health service factors. Thus, policy implication for reducing the rates of PPH in improving the health care services by ensuring the sufficient nurse midwife to patient ratio and provide sustained number of experienced nurse midwives in health care services delivery. Moreover, physical resource allocations, adequate equipment and supplies for providing basic and comprehensive emergency obstetric care in all district hospitals are important factors to consider to reduce the rate of PPH.

## Data Availability

The datasets used and/or analyzed during the current study available from the corresponding author on reasonable request.

## Abbreviations

BBA: Birth before admission
C/S: Cesarean section
EmOC: Emergency obstetric care
F/E: Forceps extraction
GDM: Gestational diabetes mellitus
ICC: Intraclass correlation coefficient
MOPH: The Ministry of Public Health
PPH: Postpartum hemorrhage
V/E: Vacuum extraction
